# How Can Unintended Pregnancies Be Prevented among Adolescents Who Engaged in Sexual Intercourse at Earlier Ages? The Role of Female Education and Partner Age Difference

**DOI:** 10.3390/ijerph182010631

**Published:** 2021-10-11

**Authors:** Raquel Pires, Anabela Araújo-Pedrosa, Joana Pereira, Maria Cristina Canavarro

**Affiliations:** 1 Center for Research in Neuropsychology and Cognitive Behavioral Intervention, Faculty of Psychology and Education Sciences, University of Coimbra, 3000-115 Coimbra, Portugal; anabela.araujopedrosa@gmail.com (A.A.-P.); joanaifpereira88@gmail.com (J.P.); mccanavarro@fpce.uc.pt (M.C.C.); 2 Clinical Psychology Service Centro Hospitalar e Universitário de Coimbra, 3030-165 Coimbra, Portugal

**Keywords:** adolescents, age at first sexual intercourse, female education, partner age difference, unintended pregnancy

## Abstract

Several studies have identified explicative factors for adolescents’ sexual risk behaviors and related outcomes such as unintended pregnancy; however, less is known about the mechanisms through which such factors act. Our study explored the role of female education and partner age difference as explicative mechanisms of the association between age at first sexual intercourse (AFSI) and unintended pregnancy while controlling for the role of other contextual factors (i.e., socioeconomic status, ethnicity, religious beliefs, and place of residence) and sexual-related mechanisms (i.e., number of sexual partners) that are known to be associated with adolescent pregnancy. The sample consisted of 613 sexually experienced female adolescents who did not intend to become pregnant: 349 were pregnant for the first time, and 264 had never been pregnant. Mediation and moderation analyses were performed. An earlier AFSI was associated with unintended pregnancy 1–6 years after first sexual intercourse by increasing the adolescents’ likelihood of having less education and being involved with partners older than themselves. There was no significant direct effect of AFSI on pregnancy occurrence after controlling for the mediators. Our findings bring to light nonsexual mechanisms that must be considered in public health interventions aimed at preventing unintended pregnancies among adolescents who engaged in sexual intercourse at early ages. Specific implications are discussed.

## 1. Introduction

Adolescent pregnancy remains a public health concern in many developed countries, namely, due to the fact of its medical, psychological, social, and financial consequences [1,2,3,4,5]. In Portugal, adolescent pregnancy rates remain above the European Union average [2,6,7]. In 2019, there were 2080 live births to Portuguese adolescent mothers, which corresponded to almost 2.5% of the total live births in the country [8]. Almost 80% of these pregnancies were not intended [4] and could have been prevented by the consistent and correct use of contraceptives or by taking compensatory actions following contraceptive failure [9,10,11].

Several studies have identified explicative factors for sexual and contraceptive risk behaviors among general samples of adolescents, producing important guidelines for the universal prevention of adolescent pregnancy [1,12]; however, the lack of resources in schools and health care centers is usually a barrier to providing preventive interventions to all adolescents [1]. Additionally, several reviews and meta-analyses have recently found insufficient evidence about the effectiveness of the existing sexual risk-reduction interventions in preventing pregnancies among general samples of adolescents [13,14,15].

Previous research has suggested that selective interventions may be superior to universal interventions to prevent adolescents’ risk behaviors and related outcomes, such as unintended pregnancies. Selective interventions are designed and implemented according to the specific characteristics and needs of adolescents at greater risk for unintended pregnancy and these allow us to address specific patterns and pathways of risk, as well as hypothesize antecedents of risk behavior change for a particular population [16,17,18,19]. However, to support the development of selective interventions aimed at preventing adolescent pregnancy, it is important to gain knowledge on the interplay between risk factors when predicting unintended pregnancies, namely, considering both female and couple-related variables [11], a topic that has seldom been studied [1]. This knowledge would contribute to clarifying how to intervene to prevent unintended pregnancies among specific risk groups of adolescents, possibly increasing both the efficacy and efficiency of adolescent pregnancy prevention.

In Portugal, as in other developed countries, recent data show that approximately 98% of adolescent pregnancies occur in the context of an intimate relationship (vs. occasional sexual encounters), at least one year after first sexual intercourse (vs. at first sexual intercourse), and usually among female adolescents who have engaged in first sexual intercourse earlier than their peers [12,18]. Based on these findings and according to a developmental psychopathology [20,21] and ecological perspective [22], the current study used a sample of sexually experienced female adolescents who were involved in an intimate relationship and focused on the interplay between three well-established predictors of adolescent pregnancy: earlier age at first sexual intercourse (AFSI), lower female education, and greater partner age difference. This was done by examining the mediating role of female education and partner age difference on the association between AFSI and the occurrence of unintended pregnancy while controlling for other contextual factors [23] and sexual-related mechanisms [24,25] commonly associated with an increased risk for adolescent pregnancy.

### 1.1. AFSI and Adolescent Pregnancy

It is known that adolescent sexual risk behavior is influenced by several developmental transitions [17]: engaging in first sexual intercourse is one of them. An earlier AFSI has been consistently identified as the first chronological factor related to adolescents’ sexual lives that increases the risk of adolescent pregnancy, particularly for females [1,25,26].

During the last few decades, several preventive programs have specifically targeted early first sexual intercourse among general and high-risk samples of adolescents. Most of the programs were designed to delay sexual initiation/promote sexual abstinence and hinged on the premise that delayed sexual intercourse would allow adolescents to eventually achieve social, cognitive, and moral maturity to successfully manage the complexities and risks of sexual interactions [16]. Then, adolescents would be able to avoid other sexual behaviors that have been traditionally used to explain the association between an earlier AFSI and adolescent pregnancy based on a risk-taking perspective (e.g., higher frequency of sexual activity and higher number of sexual partners, namely, in the context of occasional sexual encounters) [24,25]. However, the data provided thus far did not support the effectiveness of abstinence-only programs in reducing adolescent pregnancies among general samples of adolescents [13,14,27].

According to several authors, rather than focusing on the delay of sexual intercourse, future interventions should prioritize comprehensive and multifaceted approaches specifically designed for adolescents who engaged in sexual intercourse earlier than their peers [12,18,19,28,29,30,31]. These approaches should take into consideration the specific patterns and pathways of risk presented by these adolescents [17,29,30,31], considering both sexual and nonsexual factors from different ecological levels of adolescents’ lives [19,28]. However, the lack of studies about these topics makes it difficult to develop such interventions. To our knowledge, no previous study has specifically addressed nonsexual mechanisms by which an earlier AFSI may lead to an increased risk of unintended pregnancy during adolescence.

### 1.2. The Role of Female Education and Partner Age Difference

The developmental psychopathology perspective has been widely utilized to guide adolescent pregnancy prevention research and constitutes the conceptual framework of the present study [12,18,20,21,22,29]. Although the value of the research provided thus far is undeniable, from a public health perspective, it may be particularly important to gain knowledge on the developmental patterns of adolescents who engaged in first sexual intercourse at earlier ages by examining them within the main ecological contexts where their knowledge, abilities, skills, and attitudes are expected to be built and/or consolidated (e.g., school). The interpersonal contexts in which sexual risk behaviors may occur (i.e., intimate relationships) must also be clarified to guide preventive efforts [11,16,29,31,32].

Several studies have suggested associations between an earlier AFSI and two strong predictors of adolescent pregnancy that are related to the aforementioned contexts: lower female education and greater partner age difference. First, although lower educational achievement and a lack of school connectedness have been traditionally analyzed as risk factors for earlier AFSI [18], several studies found that adolescents who engaged in sexual intercourse earlier than their peers also reported a subsequent reduced interest in academic activities and objectives and, thus, demonstrated an increased likelihood of dropping out of school and being less educated [1,33,34]. Additionally, the association between less education and adolescent pregnancy is well established and has been found to be twofold [1]: it has been linked to a lack of knowledge [35] and to lower cognitive ability, which decrease one’s competence in contraceptive decision-making and management [36]; it has also been traced to an absence of academic aspirations, which is, in turn, associated with a decrease in the perceived impact of becoming pregnant during adolescence [1,37].

Second, although partner age difference has also been traditionally analyzed as a risk factor for earlier AFSI [38], adolescents who become pregnant usually report having had multiple sexual partners before pregnancy [25] and become pregnant on average one to two years after first sexual intercourse (vs. at first sexual intercourse) [13] by men older than themselves [39]. These findings suggest that adolescents who become pregnant chose new partners or continue to be involved with partners older than themselves after their first sexual intercourse. Accordingly, several authors have stated that adolescents develop elaborate ideas concerning sexuality and their sexual roles that determine which types of sexual behaviors and partners are appropriate and that an earlier AFSI may increase the likelihood of less normative sexual choices [40,41] such as pursuing sexual relationships with older adolescents, young adults, or adults [1]. In turn, research has consistently shown that engaging in sexual intercourse with males older than themselves increased adolescents’ risk of pregnancy [1,38,42]. Partner age difference is a relevant link between distal predictors (e.g., perceived physical maturity) and multiple types of risky behaviors, including pregnancy-risk behaviors [1,43,44]. Because these partners are usually adults [45] or older adolescents who have dropped out of school [46], they may perceive a lower impact of having a baby [47]. Additionally, early female adolescents may not have the communication and negotiation skills needed to engage in pregnancy prevention behaviors, particularly with older and more experienced partners [1,38].

### 1.3. The Present Study

The present study was planned in accordance with previous research that suggested that selective interventions may be superior to universal interventions to prevent adolescent risk behaviors and related outcomes [16,17,18,19]. In addition, the present study attended to the importance of knowledge about the interplay between nonsexual and sexual-related factors and mechanisms that may explain unintended adolescent pregnancies [19,28] to guide the development of public health selective interventions aimed at preventing this specific outcome in a more effective and efficient way.

Specifically, our aim was to explore whether the association between AFSI and unintended pregnancy may be explained by female education and partner age difference. Although we were particularly interested in clarifying the role of nonsexual explicative mechanisms on the association between AFSI and unintended pregnancy, attending to the role that sexual-related behaviors may have on this association [24,25], our conceptual model was tested only among adolescents involved in intimate relationships (vs. occasional sexual encounters) and considered the expected indirect effect of AFSI on unintended pregnancy through the number of sexual partners. We hypothesized that an earlier AFSI would be associated with unintended pregnancy by increasing adolescents’ likelihood of having less education and being involved with partners older than themselves, even if the number of sexual partners also mediated the association between AFSI and pregnancy occurrence.

Considering the dynamic temporal nature of two of the mediators (i.e., female education and number of sexual partners) and the presumed direct effect of AFSI on pregnancy occurrence—as early AFSI increases the period over which adolescents are at risk for pregnancy [18]—we also explored whether the time that had passed since first sexual intercourse influenced the respective indirect and direct effects of AFSI on unintended pregnancy. We hypothesized that the mediating role of female education and number of sexual partners on the association between AFSI and unintended pregnancy, as well as the direct effect of AFSI on unintended pregnancy, may increase as the time that had passed since first sexual intercourse increases. We did not have reasons to expect that the same may be true regarding partner age difference [25,39,40,41].

**Figure 1:**
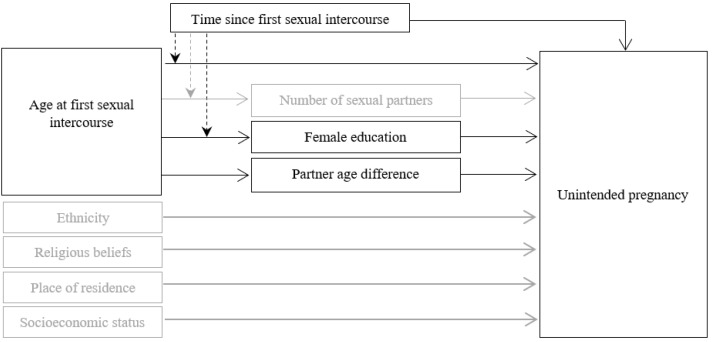
Conceptual model for the presumed association between age at first sexual intercourse (AFSI) and unintended pregnancy through female education and partner age difference while considering for: (1) the expected mediating role of number of sexual partners on the association between AFSI and unintended pregnancy, (2) the direct effects of ethnicity, socioeconomic status, religious beliefs, and place of residence on unintended pregnancy, and (3) the presumed moderation of the time that had passed since first sexual intercourse on the associations between AFSI and female education and between AFSI and number of sexual partners.

Finally, according to previous research regarding the sociocultural and socioeconomic factors associated with unintended pregnancy among adolescents [6,12,23], the study hypotheses were tested while controlling for the expected influence of ethnicity, religious beliefs, place of residence, and socioeconomic status on unintended pregnancy. The full conceptual model tested in the present study is presented in Figure 1.

## 2. Materials and Methods

### 2.1. Data Collection

This cross-sectional study is part of a larger Portuguese research project entitled “Adolescent pregnancy in Portugal: Etiology, reproductive decision, and adjustment”. Prior to its implementation, this research project was approved by the Institutional Review Board of the Faculty of Psychology and Educational Sciences, University of Coimbra, Portugal. The information described in this section and additional details regarding the project materials and methods are also available in previous related articles [1,4,6,18,23]. Data collection for this project occurred between 2008 and 2018 at 48 public health services and 22 public schools. The institutions where the sample was collected were selected to reach a representative sample of the adolescent population living in each Portuguese region. Specifically, the health care centers chosen provided central health services in their respective regions. In Portugal, this means that adolescents from the whole region are usually treated and/or routinely followed in these centers. Schools were randomly selected in each region, and the sample collection took place in those in which we secured permission to conduct our study. The sample collection was approved and carried out in compliance with ethical standards from all the health and educational services ethics committees where the adolescents were recruited [6,18,23]. The eligibility criteria for inclusion were being female, being between 10 and 19 years old [48], and having the ability to understand the questionnaire. Adolescents recruited through public health services were invited to participate during medical appointments. Adolescents recruited from public schools were invited to participate during class time. Detailed information about the study’s aims and procedures was provided to all potential participants. Written informed consent was obtained from all participants aged 18 and older and written informed assent and parental consent were obtained for participants who were minors. After providing written informed assent and consent, all participants completed a paper–pencil questionnaire in the presence of a research assistant [23]. Informed assent and consent were stored with no possible connection with patients’ responses to the study questionnaire. The anonymous nature of the data is irreversible. A total of 1231 female adolescents participated in this research project.

### 2.2. Study Sample

**Figure 2:**
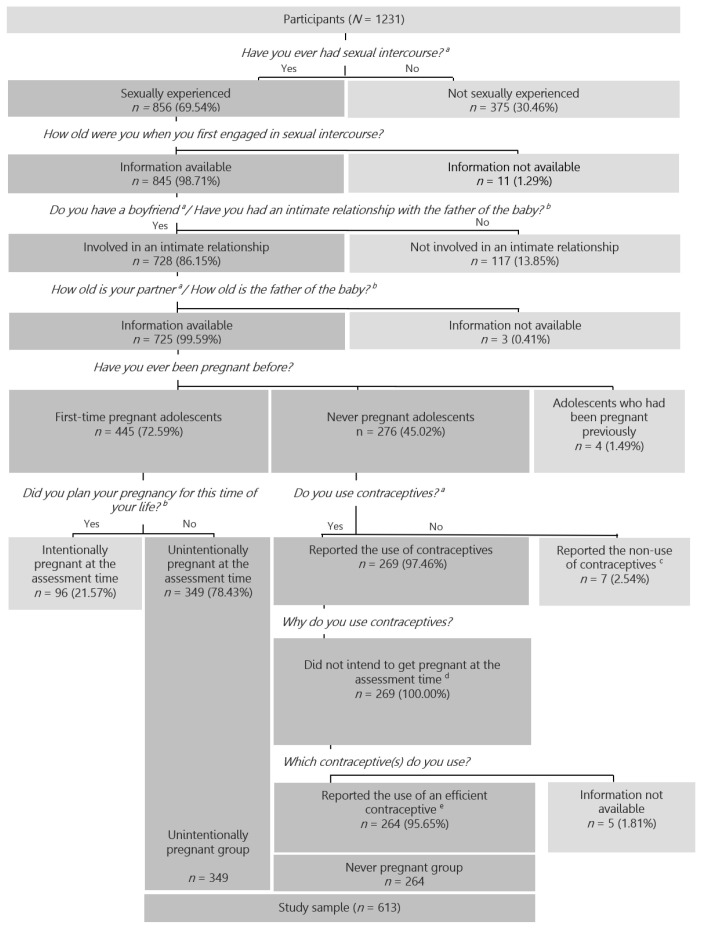
Sample selection. Note. The order of the questions in the figure follows the order by which the authors selected the study sample from the global database; this order was not the one by which the questions were presented to the adolescents. ^a^ Questionnaire version: Non-pregnant adolescents. ^b^ Questionnaire version: Pregnant adolescents. ^c^ Reporting the non-use of contraceptives due to the absence of risk of becoming pregnant (i.e., not being currently sexually active, being homosexual or having an infertile partner). ^d^ The reasons for using contraceptives were used as proxies for pregnancy intention (e.g., “I want to avoid pregnancy”, “I’m too young to be a mother”, “I am not ready for children yet”). ^e^ Condom, pill, implant, diaphragm, and/or vaginal ring.

The sample examined in the present study was selected from the total sample described above (Figure 2) and included 613 sexually experienced female adolescents, aged 12–19, who were involved in intimate relationships and who did not intend to become pregnant: 349 were pregnant for the first time at study assessment (unintentionally pregnant adolescents) and 264 had never been pregnant and reported the use of an effective contraceptive (never pregnant adolescents). Exclusion criteria for the present study and the number of adolescents excluded according to each one of them are described in Figure 1.

### 2.3. Measures

Data were obtained through a self-report questionnaire developed by the researchers [49]. As described in previous related articles [6,18,23], study questions were selected from the clinical assessment interview for pregnant adolescent patients used by the Psychological Maternity Intervention Unit at the University of Coimbra Hospitals [49]. This clinical interview was developed based on exhaustive international literature reviews and studies that focused on the contexts in which adolescent pregnancy usually occurs [50,51]. In the final version of the questionnaire, questions had two equivalent versions: one version adapted to adolescents who were pregnant at the time of the assessment and the other version adapted to adolescents who were not pregnant at the time of the assessment. The questionnaire was pilot-tested and adjusted to ensure clarity, comprehension, and suitability and has been used in several studies using samples of Portuguese pregnant adolescents [4,6,12,18,23,42,52,53,54,55,56].

#### 2.3.1. Sample Selection Variables

The questionnaire included questions regarding sexual and reproductive variables that were used for sample selection purposes: first sexual intercourse (“Have you ever had sexual intercourse?”; yes or no response) and pregnancy history (“Have you ever been pregnant?”; response options: “Yes, I am pregnant”; “Yes, I have been pregnant before”; “No, I have never been pregnant”). Adolescents who were not pregnant at the time of the assessment (“If you are not pregnant”) were also asked about contraceptive use (“Do you use contraceptives?”: yes or no response; “Which contraceptives do you use?”; “Why do you use contraceptives?”). As in our most recent study [23], adolescents who reported the use of condoms, pills, implants, diaphragms, and/or vaginal rings were considered to use an efficient precoitus contraceptive. The reasons for using contraceptives were used as proxies for pregnancy intention (e.g., adolescents who responded, “I want to avoid pregnancy”, “I am too young to be a mother”, and/or “I am not ready for children yet” were considered not intending to become pregnant. Adolescents who were pregnant at the time of the assessment (“If you are pregnant”) were asked about pregnancy intention (“Did you plan your pregnancy for this time of your life?”; yes or no response) [23].

#### 2.3.2. Study Variables

The dependent variable for the present study was defined as unintended pregnancy (0 = No, 1 = Yes). Adolescents who did not intend to become pregnant but were pregnant for the first time at the study assessment were included in the “unintentionally pregnant” group. Adolescents who did not intend to become pregnant, reported the use of an effective contraceptive, and had never been pregnant were included in the “never pregnant” group (Figure 1). The independent variable, AFSI, was assessed with the question, “How old were you (in years) when you first engaged in sexual intercourse?”. Regarding the mediators, female education, number of sexual partners, and the age of current sexual partners were assessed with the questions, “What is your grade level (years of education completed)?”, “How many people have you had sex with?”, and “How old (in years) is your partner (not pregnant adolescents)/the father of the baby (pregnant adolescents who reported an intimate relationship with the father of the baby)?”. Partner age difference (in years) was computed by subtracting the female’s age from her partner’s age. The moderator was defined as the time since sexual initiation (in years) and computed by subtracting the AFSI from female’s current age.

#### 2.3.3. Covariables

Questions for characterization and covariance control were also included, namely, those regarding other contextual factors usually associated with adolescent pregnancy [6,12,18,23]: female age (in years), religious beliefs (“Do you have any religious beliefs?”; yes or no response), place of residence (“Where do you live (district)?”), ethnicity, and socioeconomic status. Place of residence was coded based on the number of inhabitants/km^2^ of the reported living location (0 = urban (population density > 500 inhabitants/km^2^), 1 = suburban and urban (population density ≤ 500 inhabitants/km^2^)) [23,57]. For ethnicity, participants were asked to select one of the following options: European, African, Asian, Latina, or Romani. Given the adolescents’ distribution in the above categories, this variable was dummy coded as European/non-European origin [23]. Adolescents’ socioeconomic status was assessed by considering 2 additional questions that asked about the educational level and occupation of the family’s main provider (low (e.g., nonspecialized workers), medium (e.g., small business owners, high school teachers), and high (e.g., governmental or private company administrators, lawyers)) [23,58].

### 2.4. Data Analyses

Analyses were conducted using the Statistical Package for the Social Sciences, v25.0 (IBM Corp. in Armonk, New York, NY, USA). Descriptive statistics and comparison analyses (*t*-tests and chi-square tests) were performed for characterization purposes. Pearson’s correlations were performed to test the associations between the study variables. Contextual variables, other than the study variables, that showed significant associations with unintended pregnancy or at least one of the mediators were included as covariates in further analyses.

A computational tool for mediation analysis was used to test the indirect effects of AFSI on unintended pregnancy through female age, partner age difference, and number of sexual partners (PROCESS procedure for SPSS, v3.5.2; parallel mediation model; PROCESS Model 4 [59]. In addition to estimating the model coefficients using maximum likelihood logistic regression, this computational tool generated direct, single indirect, and total indirect effects in the mediation models with multiple mediators operating in parallel. Bootstrapping (*n* = 5000 samples) with bias-corrected and accelerated confidence intervals was used. When the bootstrapped 95% confidence interval (CI) of the point estimate did not include zero, the effect was significant [59].

To test whether the indirect effects of AFSI on pregnancy occurrence through female education, partner age difference, and number of sexual partners were different over time (i.e., varied according to the number of years that had passed since first sexual intercourse), a mediation-moderated model was tested (PROCESS procedure for SPSS, v3.5.2; Model 8) [59]. The covariates that had an explicative role in the mediators or in the outcome in the parallel mediation model were considered for moderation analysis. All the mediators were included, independent of the significance of their previous indirect effect (as the presence of the moderator may contribute to clarifying if a previous nonsignificant effect may become significant under specific circumstances). The conditional indirect effects of female education, partner age difference, and number of sexual partners were analyzed considering the 95% CI.

## 3. Results

### 3.1. Sample Characteristics and Bivariate Analysis

The study sample comprised 613 female adolescents, with a mean age of 16.77 years (SD = 1.27). The global mean years of the adolescents’ education was 9.41 (SD = 2.24). The adolescents were predominantly of European ethnic origin (*n* = 561, 91.5%), Catholic or followers of another religion (*n* = 464, 75.7%), and of low socioeconomic status (*n* = 508, 82.9%). Most of them were living in urban places (*n* = 462, 75.4%). The adolescents engaged in sexual intercourse for the first time between the ages of 11 and 19 (*M* = 15.17; SD = 1.02), 1–6 years before study assessment (*M* = 1.77; SD = 1.02), and had 1–10 sexual partners (*M* = 1.61, SD = 0.96). At the time of study assessment, they were involved with partners 2 years younger to 23 years older than themselves (*M* = 3.25, SD = 3.11). Detailed descriptive statistics for the total sample and comparisons between study groups are presented in Table 1.

Pearson’s correlations for all variables are presented in Table 2. Being younger, of non-European ethnic origin, with no religious beliefs, and having a low socioeconomic status were variables that were significantly associated with unintended pregnancy. Engaging in sexual intercourse at earlier ages, having less education, and being involved with partners older than themselves were also associated with unintended pregnancy.

### 3.2. Parallel Mediation Model

**Figure 3:**
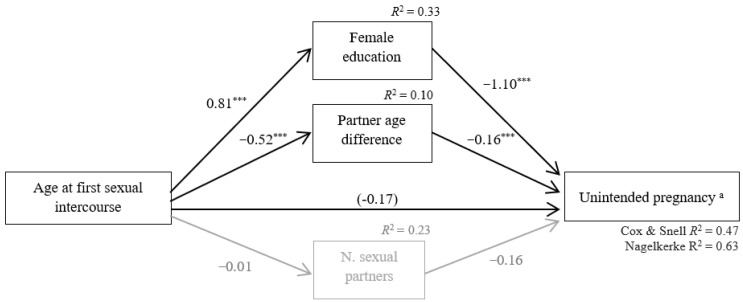
Statistical diagram of the parallel mediation model for the presumed association between age at first sexual intercourse (AFSI) and unintended pregnancy through female education and partner age difference and number of sexual partners while controlling for ethnicity, socioeconomic status, religious beliefs, place of residence, and time since sexual initiation. Path values represent unstandardized regression coefficients. In the arrow linking AFSI and unintended pregnancy, the value inside parentheses represents the direct effect of AFSI on unintended pregnancy after controlling for the mediators. The total effect of AFSI on unintended pregnancy is not available because unintended pregnancy is a dichotomous variable. The significant indirect effects are represented by paths in black. Non-significant indirect effects are represented by paths in gray. Significant mediators: female education and partner age difference. Non-significant mediators: number of sexual partners. Note. *N* = 613. ^a^ Reference category: No. *** *p* < 0.001.

Significant indirect effects of AFSI on pregnancy occurrence through female education (point estimate = −0.91, CI = −1.21/−0.70) and partner age difference (point estimate = −0.09, CI = −0.16/−0.03) were found. The indirect effect of AFSI on unintended pregnancy through the number of sexual partners was not significant (point estimate = 0.00, CI = −0.01/0.02). All paths for the indirect effects, as well as unstandardized regression coefficients, are illustrated in Figure 3. As depicted, an earlier AFSI increased the adolescents’ risk of pregnancy by increasing their likelihood of having less education and being involved with partners older than themselves. There was no significant direct effect of AFSI on pregnancy occurrence after controlling for the mediators (point estimate = −0.17, CI = −0.41/0.08).

The parallel mediation model was significant (–2 loglikelihood = 449.35; X^2^(9) = 388.62, *p* < 0.001) and explained from 47% (Cox and Snell R^2^) to 63% (Nagelkerke R^2^) of the variance in unintended pregnancy. All the covariates were significantly associated with at least one mediator or with the outcome variable (data not shown).

### 3.3. Moderated Mediation Model

The time that had passed since first sexual intercourse was found to be a significant moderator of the indirect effects of AFSI on unintended pregnancy through female education and number of sexual partners but not through partner age difference. The interactions between AFSI and time since first sexual intercourse were only significant when predicting female education and number of sexual partners. Post hoc analyses were used to determine the nature of the conditional indirect effect of AFSI on unintended pregnancy via female education at different values of the moderator and showed that the indirect effect remained significant for all values of the moderator but increased as time since first sexual intercourse increased. The post hoc analyses used to determine the nature of the conditional indirect effect of AFSI on pregnancy occurrence via the number of sexual partners at different values of the moderator showed that the indirect effect remained nonsignificant for all values of the moderator. The moderated mediation model is fully described in Table 3.

## 4. Discussion

Our aim was to explore the indirect effects of AFSI on unintended pregnancy among female adolescents involved in intimate relationships by examining whether these effects were related to the variables of female education and partner age difference. Overall, our results support the proposed effects, as an earlier AFSI increased the adolescents’ risk of unintended pregnancy 1–6 years after sexual initiation by increasing their likelihood of having less education and being involved with partners older than themselves. As discussed below, these findings have important implications for practice and research in adolescent pregnancy prevention.

Our findings are consistent with previous research that suggested a negative association between AFSI and female education and a higher risk of pregnancy among adolescents with less education [1,33,35,36,37]. We also found evidence supporting previous research regarding the negative association between AFSI and partner age difference and a higher risk of pregnancy among adolescents involved with partners older than themselves [1,25,38,39,40,41,42]. Moreover, the current study adds to previous research, showing that, regardless of socioeconomic status, ethnicity, religious beliefs, place of residence, number of sexual partners, and time passed since first sexual intercourse, female education and partner age difference contributed to explaining the association between females’ AFSI and unintended pregnancy, which are important aspects to consider in the development of future research and public health interventions in the field.

The association between an earlier AFSI and adolescent pregnancy has been traditionally explained as part of a sexual risk-taking pathway involving other sexual risk behaviors such as a higher frequency of sexual activity, namely, in the context of occasional sexual encounters, and a higher number of sexual partners [24,25]. This pathway has also been associated with individual characteristics such as attraction to risk, under control, or sensation-seeking [18,24,25,40]. However, our findings bring to light other factors that should be considered when planning future research in the field and public health interventions aimed to prevent pregnancy among adolescents who engaged in first sexual intercourse at earlier ages. The association between AFSI and unintended pregnancy through female education and partner age difference was found in a sample exclusively composed by adolescents involved in a romantic relationship (vs. occasional encounters) while attending to the potential role of the number of sexual partners and time passed since first sexual intercourse. These findings suggest that explanations of the association between AFSI and unintended pregnancy based on sexual risk-taking pathways or greater time of risk exposure may be valid but are not exclusive.

Regarding female education, a possible explanation of our findings is that the lack of knowledge and the lower cognitive ability usually associated with lower educational levels may decrease adolescents’ competences in contraceptive decision-making and management [35,36]. Additionally, the absence of academic aspirations, usually also linked to lower educational levels, may decrease the perceived impact of becoming pregnant during adolescence and increase contraceptive risk behaviors [37]. Moreover, if adolescents who engage in sexual intercourse at earlier ages tend to achieve lower educational levels, they may have less access to sexual education initiatives adequate for their physical, cognitive, and emotional development. First, if they dropped out of school at early ages, they would not have access to the continuous sexual education provided in this setting in most developed countries. Second, sexual education is usually adjusted to the normative ages of students in each class. This may exclude the developmental needs and specific characteristics of the older students with one or more years of school retention. Although these adolescents may be older and sexually more experienced than their class peers, they may have a lower ability to understand, integrate, and applicate the information given to them or the skills that they developed during sexual education classes. Our findings stress that preventing academic failure and fostering the pursuit of educational goals, in conjunction with decision-making and contraceptive management support adapted to the knowledge, cognitive ability, reproductive and emotional development and sexual experience of the at-risk adolescents, performed both inside and outside the school environment, may be of particular importance to preventing unintended pregnancies among adolescents who engaged in sexual intercourse at earlier ages.

Regarding partner age difference, our findings were consistent with the hypothesis that AFSI increased the risk of unintended pregnancy by increasing the adolescents’ likelihood of being involved with older adolescents, young adults, or adults [1]. These findings highlight the need to teach at-risk adolescents communication skills earlier that may allow them to effectively negotiate pregnancy prevention with partners older than themselves. Further research that allows us to easily identify adolescents with a high probability of engaging in sexual intercourse earlier than their peers may be useful at this point. According to our findings, from the age of 11, some adolescents should be able to engage in quality negotiation processes with males older than themselves. Interventions should target females’ perceptions of several topics, namely, support from their partners regarding contraceptive use, relationship power, and females’ and partners’ pregnancy intentions [1,10]. Empowering these adolescents to clearly communicate about pregnancy intentions with their partners, to make informed choices about contraception and to seek professional support at school or at health care centers whenever they feel that their contraceptive management skills are not in line with their pregnancy intentions may be also important. Involving adolescents’ partners in family planning efforts before and after first sexual intercourse, in a non-judgmental way, may also be useful. Providers and educators also need to adapt their communication style to the individual and contextual characteristics of these adolescents and their partners. Further research on partners’ characteristics is needed to efficiently identify and target at-risk groups of males for sexual education. According to our findings, among pregnant adolescents, partner age difference ranged from −2 to 23 years, indicating that some male partners were adults. This may help to explain the insufficient results observed in several preventive programs provided exclusively at school and/or that focus on peer-aged couples, highlighting the need to rethink health policies and public health interventions to include the male population at greater risk of being involved in an adolescent pregnancy [1].

Some limitations of our study must be acknowledged. Temporal ordering was considered when selecting the variables to include in the mediation models, and the time that had passed since first sexual intercourse was also considered, but the present study had a cross-sectional design. Although developmental psychopathology researchers agree that to understand the basic processes of change involved in the relations between individuals and contexts, descriptive and exploratory research must be conducted within the actual ecology of people’s lives [31], longitudinal designs are superior for establishing timelines that allow for drawing causal inferences and should be used in future research. Furthermore, assessing variables as sensitive as AFSI, number of sexual partners and contraceptive use through single-instance and self-report measures make controlling for socially desirable answers challenging [60].

Despite these limitations, to the best of our knowledge, our study is the first to address some mechanisms by which an earlier AFSI may lead to an increased risk of unintended pregnancy among female adolescents, considering both nonsexual and sexual-related mechanisms and other contextual factors associated with adolescent pregnancy. Based on a developmental psychopathology [20,21] and ecological [22] perspective, our study contributed to the knowledge on the developmental patterns of adolescents who engaged in first sexual intercourse at earlier ages by examining them within the multiple embedded contexts in which they live [31].

Furthermore, the study comprised several conceptual and methodological advances to previous research [1]. First, previous research analyzing the consequences of earlier AFSI traditionally used “early/late” dichotomizations of AFSI based on developmental, statistical, or legal criteria that vary across countries [25]. In contrast, by considering AFSI as a continuous variable, our study allowed for direct cross-cultural comparisons. Second, most studies have been conducted with adolescents of middle school age and older [10,25,38]. Because adolescents who become pregnant tend to initiate sexual activity and drop out of school at earlier ages [12], this methodological decision may be limited in its usefulness for adolescent pregnancy prevention. As such, we included a wide range of ages and recruited participants in settings other than school. Third, contrary to most studies [10,25,38], when analyzing pregnancy occurrence, we excluded adolescents who had intentionally become pregnant. We also avoided relevant, but less informative, comparisons between pregnant adolescents and adolescents of the general population without considering sexual and relationship experiences, pregnancy intentions, or contraceptive behaviors of the latter group [12].

## 5. Conclusions

Regardless of socioeconomic status, ethnicity, religious beliefs, place of residence, number of sexual partners, and time passed since first sexual intercourse, an earlier AFSI increased the adolescents’ risk of unintended pregnancy 1–6 years after sexual initiation by increasing their likelihood of having less education and being involved with partners older than themselves. The association between AFSI and unintended pregnancy through female education and partner age difference was found in a sample exclusively composed by female adolescents involved in a romantic relationship (vs. occasional encounters). These findings highlight the need to consider approaches other than those based on sexual risk-taking characteristics when planning public health interventions to prevent unintended pregnancies among adolescents who engage in sexual intercourse at earlier ages. Factors related to adolescents’ knowledge, physic, emotional and cognitive development, sexual experience, life expectancies, and their ability to communicate, negotiate and manage contraceptive use in the context of romantic relationships with males older than themselves—namely older adolescents, young adults, and adults—should be considered. The content of the interventions and the communication strategies used by the health care providers and educators that are conducting them must be adapted to the specific developmental characteristics of the adolescents at greater risk to be effective. The knowledge gained in this study may enable health care providers, educators, and policy makers to optimize resources and efficacy in adolescent pregnancy prevention among this specific risk group of adolescents. Further research is needed to assess the efficacy of such interventions.

## Figures and Tables

**Table 1:** Descriptive Statistics and Group Comparisons.

	Total Sample *N* = 613		Never Pregnant (*n* = 349)		Unintentionally Pregnant (*n* = 264)		Group Comparison
	M (SD)	Range	M (SD)	Range	M (SD)	Range	*t*/χ^2^
**Participants’ characteristics**							
Age	16.77 (1.27)	12–19	17.30 (1.10)	14–19	16.38 (1.25)	12–19	9.45 **
Ethnicity: European origin (*n*, %)	561	91.5	250	94.7	311	89.1	6.04 *
Religious beliefs: Yes (*n*, %)	464	75.7	230	87.1	234	67.0	32.92 **
Place of residence: Urban (*n*, %)	462	75.4	204	77.3	258	73.9	0.91
Socioeconomic status: Low (*n*, %)	508	82.9	187	70.8	321	92.0	47.34 **
**Study variables**							
Age at first sexual intercourse	15.17 (1.37)	11–19	15.74 (1.31)	13–19	14.74 (1.24)	11–18	9.66 **
Partner age difference	3.24 (3.22)	−2–23	2.25 (2.48)	−2–13	4.00 (3.33)	−1–23	−7.43 **
Female education	9.41 (2.24)	0–14	11.03 (1.18)	6–14	8.17 (2.06)	0–12	21.66 **
Time since sexual initiation (years)	1.77 (1.02)	1–6	1.73 (0.99)	1–5	1.79 (1.05)	1–6	−0.72 **
Number of sexual partners	1.61 (0.96)	1–10	1.62 (1.07)	1–10	1.61 (0.87)	1–7	0.18
Unintended pregnancy: Yes (*n*, %)	349	56.9	−	−	−	−	−

* *p* < 0.05; ** *p* < 0.001.

**Table 2:** Pearson’s Correlations for All Variables in Study.

	Correlations between All Variables					
	1.	2.	3.	4.	5.	6.
**Participants’ characteristics**						
Age (years)	0.59 **	−0.15 **	0.46 **	0.16 **	0.35 **	−0.36 **
Ethnicity ^a^	−0.05	0.09 *	−0.18 **	0.10 *	−0.03	0.10 *
Religious beliefs ^b^	0.16 **	−0.04	0.20 **	−0.06	−0.08	−0.23 **
Place of residence ^c^	−0.01	0.15 **	−0.15 **	−0.07	0.01	0.04
Socioeconomic status ^d^	0.09 *	−0.13 **	0.26 **	0.02	0.01	−0.28 **
**Study variables**						
1. AFSI	−	−0.26 **	0.40 **	−0.26 **	−0.51 **	−0.36 **
2. Partner age difference ^e^	−	−	−0.26 **	0.06	0.14 **	0.28 **
3. Female education ^f^	−	−	−	0.02	0.02	−0.63 **
4. Number of sexual partners	−	−	−	−	0.46 **	−0.01
5. Time since first sexual intercourse (years)	−	−	−	−	−	0.03
6. Unintended pregnancy ^g^	−	−	−	−	−	−

* *p* < 0.05, ** *p* < 0.01. Note. AFSI—Age at first sexual intercourse; ^a^ Reference group: 0 = European ethnic origin; ^b^ Reference group: 0 = No; ^c^ Reference group: 0 = Urban; ^d^ Reference group: 0 = Low; ^e^ Partners’ age difference was computed by subtracting the female age (in years) from her partner age (in years). Values lower than zero indicates that the female adolescent was older than her partner; ^f^ Years of completed education; ^g^ Reference group: 0 = No.

**Table 3:** Regression Results for the Moderated Mediation Model of the Association between Age at First Sexual Intercourse and Unintended Pregnancy through Female Education, Partner Age Difference and Number of Sexual Partners, and Conditional Indirect Effects at Different Values of the moderators.

	**Mediator Variable Model 1 (Predicting Female Education)** ***R*^2^ = 0.34**			
**Independent Variables**	***b***	***SE***	**Boot LLCI**	**Boot ULCI**
Constant	−0.77	1.60	−3.92	2.38
Ethnicity ^a^	−1.15	0.27	−1.17	−0.62
Religious beliefs ^b^	0.75	0.18	0.40	1.09
Place of residence ^c^	−0.78	0.18	−1.17	−0.62
Socioeconomic status ^d^	1.02	0.20	0.63	1.42
Time since first sexual intercourse (Time SS)	−1.79	0.80	−3.36	−0.21
Age at first sexual intercourse (AFSI)	0.56	0.11	0.35	0.77
AFSI × Time SS	0.17	0.06	0.06	0.28
	**Mediator Variable Model 2 (Predicting Partner Age Difference)** ***R*^2^ = 0.11**			
	***b***	***SE***	**Boot LLCI**	**Boot ULCI**
Constant	9.28	2.59	4.19	14.38
Ethnicity ^a^	1.10	0.44	0.24	1.95
Religious beliefs ^b^	−0.07	0.29	−0.63	0.49
Place of residence ^c^	1.067	0.29	0.51	1.63
Socioeconomic status ^d^	−0.64	0.33	−1.28	0.01
Time SS	1.03	1.29	−1.51	3.57
AFSI	−0.42	0.17	−0.75	−0.08
AFSI × Time SS	−0.07	0.09	−0.25	0.11
	**Mediator Variable Model 3 (Predicting Number of Sexual Partners)** ***R*^2^ = 0.24**			
	***b***	***SE***	**Boot LLCI**	**Boot ULCI**
Constant	2.18	0.74	0.73	3.63
Ethnicity ^a^	0.37	0.12	0.13	0.62
Religious beliefs ^b^	−0.02	0.08	−0.18	0.14
Place of residence ^c^	−0.10	0.08	−0.26	0.06
Socioeconomic status ^d^	0.04	0.09	−0.15	0.22
Time SS	−0.29	0.37	−1.02	0.43
AFSI	−0.09	0.05	−0.19	0.01
AFSI × Time SS	0.05	0.03	0.01	0.10
	**Dependent Variable Model (Predicting Unintended Pregnancy ^e^)** ***Cox & Snell R*^2^ = 0.47; *Nagelkerke R*^2^ = 0.63**			
	***b***	***SE***	**Boot LLCI**	**Boot ULCI**
Constant	16.82	3.03	10.88	22.76
Ethnicity ^a^	0.26	0.49	−0.71	1.22
Religious beliefs ^b^	−0.88	0.31	−1.50	-0.35
Place of residence ^c^	−0.59	0.30	−1.17	-0.01
Socioeconomic status ^d^	−0.98	0.32	−1.61	−0.35
Female education	−1.10	0.11	−1.31	−0.88
Partner age difference	0.16	0.05	0.07	0.26
Number of sexual partners	−0.17	0.14	−0.45	0.11
Time SS	−1.48	1.36	−4.15	1.18
AFSI	−0.34	0.19	−0.73	0.05
AFSI × Time SS	0.11	0.10	−0.08	0.30
**Values of the moderator Time SS ^f^**	**Conditional Indirect Effect of AFSI through Female Education**			
	Boot indirect effect	Boot *SE*	Boot LLCI	Boot ULCI
1.00	−0.80	0.13	−1.10	−0.59
1.77	−0.94	0.13	−1.26	−0.73
2.79	−1.13	0.18	−1.55	−0.85
	**Conditional Indirect Effect of AFSI through Partner Age Difference**			
	Boot indirect effect	Boot *SE*	Boot LLCI	Boot ULCI
1.00	−0.08	0.03	−0.15	−0.02
1.77	−0.09	0.05	−0.16	−0.03
2.79	−0.10	0.04	−0.19	−0.03
	**Conditional Indirect Effect of AFSI through Number of Sexual Partners**			
	Boot indirect effect	Boot *SE*	Boot LLCI	Boot ULCI
1.00	0.01	0.01	−0.01	0.02
1.77	0.01	0.01	−0.01	0.01
2.79	−0.01	0.012	−0.04	0.01
	**Conditional Direct Effect of AFSI**			
	Boot indirect effect	Boot *SE*	Boot LLCI	Boot ULCI
1.00	−0.23	0.14	−0.50	0.04
1.77	−0.15	0.13	−0.40	0.10
2.79	−0.03	0.17	−0.37	0.30

Note. *N* = 613. Unstandardized regression coefficients are reported. LL = lower limited. CI = 95% Confidence interval. UL = Upper limited. ^a^ Reference group: 0 = European ethnic origin. ^b^ Reference group: 0 = No. ^c^ Reference group: 0 = Urban; ^d^ Reference group: 0 = Low; ^e^ Reference group: 0 = No; ^f^ Values correspond to *M*−1*SD*, *M*, and *M*+1*SD* of time since first sexual intercourse.

## Data Availability

The data presented in this study are available on request from the corresponding author. The data are not publicly available due to the fact of ethical reasons.
